# Photodynamic Therapy for the Endodontic Treatment of a Traumatic Primary Tooth in a Diabetic Pediatric Patient

**DOI:** 10.5681/joddd.2014.010

**Published:** 2014-03-05

**Authors:** Giselle de Sant’Anna

**Affiliations:** ^1^Professor, Department of Pediatric Dentistry, Cruzeiro do Sul University, Sao Paulo, SP, Brazil; ^2^Professor, Department of Cariology, Cruzeiro do Sul University, Sao Paulo, SP, Brazil; ^3^Professor, Department of Pharmacology and Therapeutics Department, Cruzeiro do Sul University, Sao Paulo, SP, Brazil

**Keywords:** Endodontics, photodynamic therapy, dental traumatism.

## Abstract

Conservation of deciduous teeth with pulp alterations caused by caries or trauma is a major therapeutic challenge in pediatric dentistry. It is essential that the sanitizers used in root canal procedures perform well in eliminating bacteria. Antimicrobial photodynamic therapy (PDT) is an emerging and promising adjuvant therapy for endodontic treatment in an attempt to eliminate microorganisms persistent after chemomechanical preparation. This paper reports the case of a five-year-old male with type I diabetes mellitus, presenting the need for pulp therapy in maxillary primary left central incisor due to injury. The proposed treatment included the use of PDT for decontamination of root canals with the application of 50 μg/mL of methylene blue dye for 3-5 minutes and 40 J/cm^2^ as energy density, taking into account the need for tissue penetration and effec-tiveness of PDT inside the dentinal tubules.

## Introduction

Traumatic injuries, especially in anterior teeth, have a high prevalence^1^ and are considered serious problems because of the pulp involvement and the emotional aspect for the patient and the parents. The maintenance of primary teeth with pulp changes caused by caries or trauma is a major therapeutic challenge in pediatric dentistry because of the biological cycle of the pulp and the internal anatomy of these teeth. Therefore, the root canal sanitizers with high performance in eliminating bacterial contamination are a key to treatment success in such teeth. Most failures or unsuccessful endodontic treatments are related to the persistence of microorganisms that survive the chemo-mechanical preparation of the teeth or the medications and dressings used.^2^

**Figure 1.**
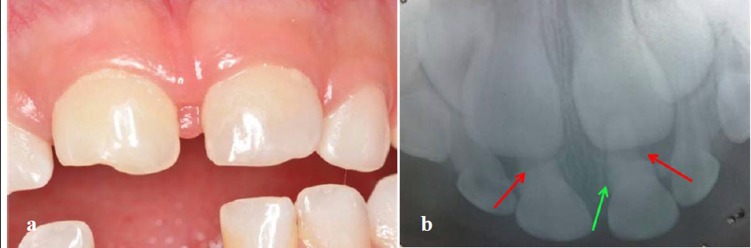
a. Clinical image of traumatism (51); enamel fracture, color change (yellow), grayish yellow change in 61 with pulp impairment. b. Radiograph with "roof of the church” resorption aspect in both elements (red arrows) and increased pericementary space (green arrow).

The pathological pulp processes are very commonly found in deciduous teeth (Figure 1a, 1b). In these processes anaerobic microorganisms were quantified in 96.7% of cases, black-pigmented bacilli (BPB) in 35.5%, aerobic in 93.5%, streptococci in 96.7% and* Streptococcus mutans *in 48.4%, constituting a polymicrobial etiology for infection.^2^

In this context, antimicrobial photodynamic therapy (PAT) is a very promising approach to disinfect dentinal walls^2^ since in the presence of oxygen found in cells, the photosensitizer activated by light can react with molecules by electrons or hydrogen transfer, leading to free radical production (type I reaction) or by energy transfer to oxygen (type II reaction), leading to singlet oxygen production. Both paths can lead to cell death, in this case, microbial disinfection.^3,4^ One significant advantage is that developing resistance to PAT by microorganisms seems unlikely since singlet oxygen and free radicals interact with various cellular structures and metabolic pathways of microbial cells.^3^ PAT is also effective against bacteria resistant to antibiotics, and repeated photosensitization has not led to selection of resistant strains.^6^

Thus, PAT has emerged as adjuvant therapy for endodontic treatment in an attempt to eliminate persistent microorganisms after chemo-mechanical preparation. Several studies have investigated PAT activity in bacteria related to pulp diseases,^2,4-6^ and the results have indicated 70% reduction of viable bacteria, with better success by combining conventional treatment and PAT.^2,4,6^

When opting for this type of therapy, some principles should be followed, including a pre-irradiation time of 3–5 minutes to sensitize the biofilm bacteria, and the use of an energy density that takes into account the characteristic of the tissue and the penetration needed for effectiveness of PDT within the dentinal tubule.

This paper reports the endodontic treatment of a deciduous tooth after dental trauma in a child with diabetes type I using PAT.

## Case report

A 5-year-old patient with insulin-dependent diabetes mellitus (type I) was referred to the Department of Pediatric Dentistry Health at Barueri, Sao Paulo, Brazil, from the Endocrinology Ward for dental evaluation. On clinical and radiographic examination, a color change was evident on teeth 51 and 61, with tooth 51 presenting an enamel fracture and tooth 61 showing great mobility (grade II) and periodontal changes (Figures 1a and 1b). On past medical history, parents reported child falling and the teeth traumatized 6–8 months prior to examination.

In radiographic examination, external resorption in a “church roof” shape was observed in both teeth, characteristic of teeth that have suffered trauma. Increased periodontal ligament (PDL) space was also observed in tooth 61. Tooth 51 demonstrated calcium degeneration (Figure 1b).

The diagnoses were color change in tooth 51 and pulpal necrosis in tooth 61, both due to trauma.

**Figure 2.**
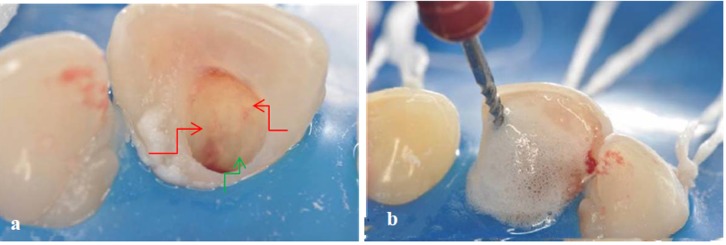
a. Root canal opened. b. Chemical and mechanical preparation using endo PTC and Milton solution.

**Figure 3.**
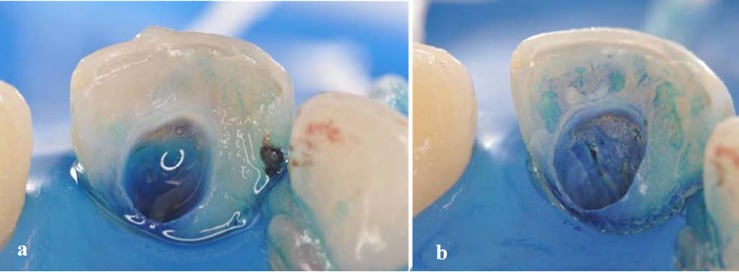
a. Use of methylene blue in solution (50 µg/mL) as a photosensitizing agent and pre-irradiation time of 5 minutes. b. Dentin tissue stained with photosensitizing agent. It is important to aspirate the contents prior to irradiation with light.

**Figure 4.**
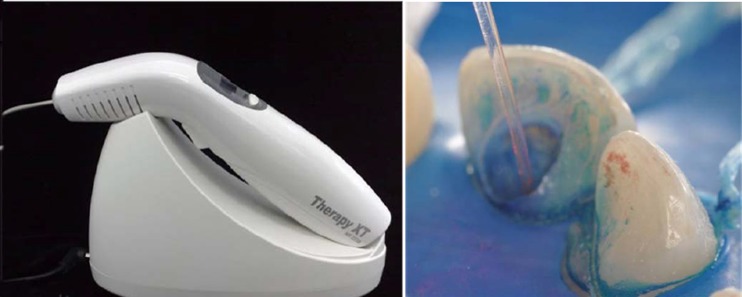
a. Laser unit used (λ=660 nm) and optical fiber (Therapy XT-DMC, São Carlos-Brazil). b. Fiber in position and irradiation (40 J/cm^2^).

**Figure 5.**
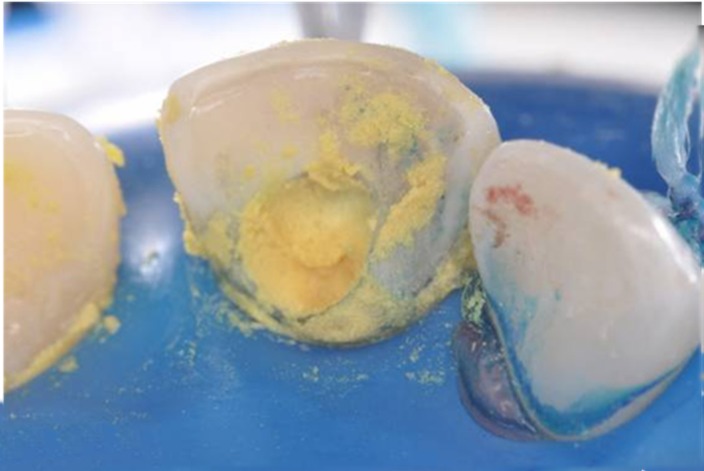
Root canal filling with iodoform paste.

**Figure 6.**
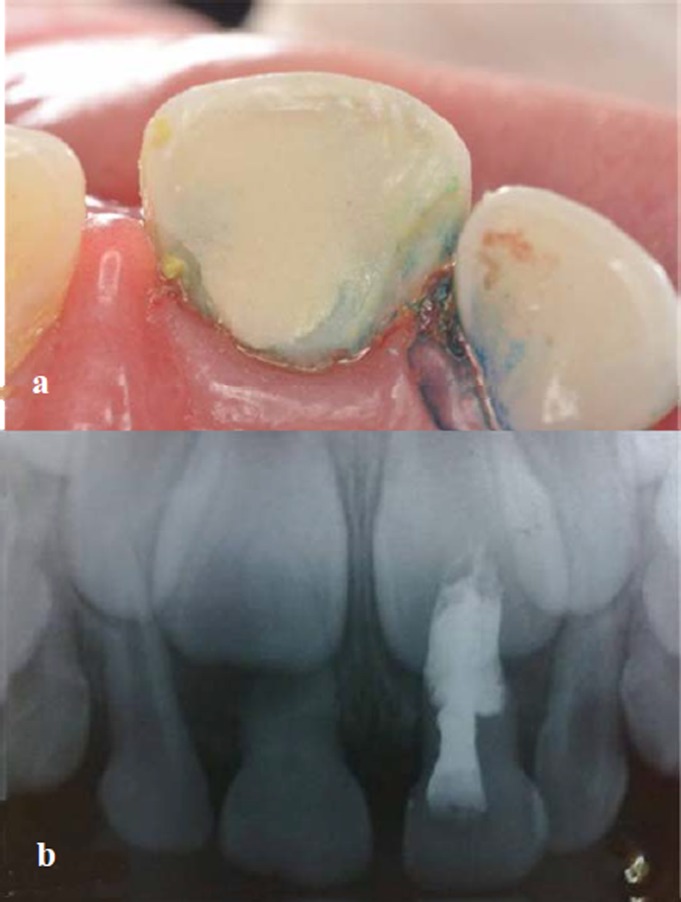
a. Temporary restoration with glass-ionomer cement. b. Final radiographic image. Note extravasation of resorbable paste for external resorption.

After preparing the access to the root canal (Figure 2a), chemo-mechanical preparation (Figure 2b) was performed using an auxiliary substance (endo PTC; Biodinâmica Química e Farmacêutica LTDA, Ibiporã, Brasil) and Milton solution (Biodinâmica Química e Farmacêutica LTDA, Ibiporã, Brasil). The tooth was prepared with files #55, #60, #70 and #80 (Figure 2b). Due to the patient’s baseline condition and the need for an effective disinfection of root canal system, PAT was used as adjunct therapy. To this end, the canal was filled with methylene blue in water solution (50 µg/mL) as photosensitizer substance and pre-irradiated for 5 minutes (Figure 3a). The content of the canal was aspirated prior to irradiation with light (Figure 3b). Using a laser unit with an optical fiber (λ = 660 nm; Figure 4a), light irradiation was performed with an energy density of 40 J/cm^2^ (Figure 4b). After root canal disinfection with both techniques, the canal was filled with iodoform paste (Figures 5,6aand6b).

## Discussion

Diabetes mellitus has a chronic and complex nature, with vascular and metabolic components. This chronic disease results from the relative or absolute insufficiency of insulin, caused either by low insulin production in pancreas or the lack of response in peripheral tissues to insulin, altering the metabolism of carbohydrates, lipids and proteins.^9^

It is believed that the frequency of infections in well-controlled diabetics is not significantly higher than that observed in the general population. On the other hand, it is known that the immune response is altered due to physiological changes, so that there is a higher susceptibility. Leukocytes exhibit lower chemotaxis and decreased motility, resulting in lower phagocytosis of infectious particles. The control of glucose relates to the etiopathogenesis of these changes, and insulin deficiency causes a reduction in the number of osteoblasts, reducing the ability of tissue repair.^10^

Given the uncontrolled blood glucose in the present case, the treatment plan included an endodontic therapy combining conventional methods of root canal disinfection with PAT in an attempt to eliminate as many bacteria from the root canal as possible.

Many types of lasers have been used for root canal disinfection, but only wavelengths are applicable, which can deliver their power through extremely fine flexible fiber optic systems (Figures 4a and 4b) and penetrate dentin to a depth that can eliminate bacteria. Laser light with a wavelength near the infrared range is absorbed by dentin only to a small extent and does not penetrate deep into the intertubular tissue in order to produce a sufficient bactericidal effect in deep layers.^8^ Photodynamic therapy included in this context can be applied as a potentially bactericidal adjunct to conventional treatment.^4-6^ In the establishment of protocols employed in PAT in endodontics, we highlight the association of laser in the red spectrum with blue photosensitizers since this type of light has a role in bone repair in the presence of periapical pathology in permanent teeth and in the furcation area in deciduous teeth, increasing bone repair associated with radicular dentin decontamination.^7^

As noted previously, several studies have investigated the performance of PAT with significant reductions in viable bacteria and better success by combining conventional treatment and PAT.^2,4-6^ The photosensitizing agent can be used in the pharmaceutical form of a solution as used in this case, or aqueous gel. It should be emphasized that in both forms the application can be processed easily. However, it is evident that the tissue is more thoroughly impregnated with dye in solution form and its removal prior to laser irradiation is easier.

It is worth noting that treatment success in pediatric dentistry is specially tied to behavior management, which aggravates with increasing operative time, and therefore, the use of a light source as an adjuvant therapy that allows for a shorter treatment session can increase the success of endodontic therapy which per se corresponds to a lengthy procedure.

Modern laser technology and the associated therapies have brought considerable advantages to successful techniques, beyond those of conventional endodontic therapy. PAT provides an excellent prognosis with substantial bacterial reduction and an interesting time–cost-and-benefits relation in pediatric dentistry.
